# Increased Plasma Superoxide Radical in Patients with Non-Metastatic Colorectal Cancer

**DOI:** 10.4021/gr2008.11.1249

**Published:** 2008-11-20

**Authors:** Stelios F. Assimakopoulos, Konstantinos Grintzalis, Ioannis Papapostolou, Konstantinos C. Thomopoulos, Christos D. Georgiou

**Affiliations:** a Department of Internal Medicine, University Hospital of Patras, Patras 26504, Greece; b Section of Genetics, Cell and Developmental Biology, Department of Biology, University of Patras, Patras, Greece; c Division of Gastroenterology, Department of Internal Medicine, University Hospital of Patras, Patras 26504, Greece

**Keywords:** colorectal cancer, oxidative stress, superoxide radical, lipid peroxidation

## Abstract

**Background:**

Several studies have investigated the potential role of oxidative stress in the evolution of colorectal cancer. In most of these studies, oxidative stress was assessed indirectly by measurements of indices like lipid peroxidation, protein oxidation or antioxidant status. The present study was undertaken to directly assess systemic oxidative stress by measuring plasma superoxide radical (O_2_^-^·) in patients with non-metastatic colorectal cancer.

**Methods:**

Twelve patients (6 males and 6 females) with a recent diagnosis of colorectal cancer and no signs of metastases and 12 healthy volunteers matched for age and gender were enrolled in the study. O_2_^-^· levels in plasma were assessed by application of a new ultra-sensitive fluorescent assay. Also lipid peroxidation levels in plasma were measured as thiobarbituric acid reactive species (TBARS).

**Results:**

In the plasma fraction of whole blood, there was a significant increase (47%) of O_2_^-^· levels in colorectal carcinoma patients as compared to healthy volunteers (P < 0.001). In fractionated plasma, no O_2_^-^· was detected in both groups. Plasma TBARS levels were increased by 81% in colorectal carcinoma patients as compared to controls (P < 0.001).

**Conclusions:**

These data show that colorectal cancer, even at early (non-metastatic) stages, induces systemic oxidative stress as evidenced by increased O_2_·^-^ levels measured in plasma. Given the important role of oxidative stress in carcinogenesis and the fact that O_2_·^-^ is considered its primary parameter, our findings if confirmed in larger studies might establish the potential validity of O_2_·^-^ as a new biomarker for colorectal cancer.

## Introduction

Oxidative stress has been implicated in a variety of both physiological (e.g. aging, differentiation, development, reproduction, cell cycle, apoptosis) and pathological processes (e.g. cancer, atherosclerosis, hypertension, diabetes, ischemia/reperfusion injury)(1). Several studies have addressed its significant role as related to the classic concepts of initiation, promotion and progression of carcinogenesis(2). Whether the tumorigenic effect of chronic oxidative stress depends on random mutations induced by reactive oxidative molecular species, or it is the result of some fragile genomic loci that are sensitive to oxidative damage in association with changes of transcriptional activity or other topologic or non-topologic effects remains to be explored.

Several studies have addressed the importance of oxidative stress in the evolution of colorectal cancer, demonstrating high oxidative stress either locally in the tumour area or systemically (3-6). In most of these studies, oxidative stress was demonstrated indirectly by increased lipid peroxidation and protein oxidation, decreased antioxidant substances and thiol groups, or decreased activity of antioxidant enzyme systems.

Superoxide radical (O_2_·^-^), resulting from the acceptance of one electron by molecular oxygen, is considered as the primary parameter of oxidative stress. Its formation *in vivo* in the blood of patients is of great clinical interest, because it directly shows the presence of oxidative stress. Superoxide’s *in vivo* measurement, which has been problematic due to its short lifetime (∼ 1 microsecond) and its continuous detoxification by intra- and extracellular antioxidants (1, 7), can now be reliably done by application of a new ultrasensitive fluorescent assay developed by our team (7, 8).

The present study was undertaken to investigate directly oxidative stress in the blood of patients with early (non-metastatic) colorectal cancer by measuring O_2_^-^·.

## Methods

### Patients

Twelve patients (6 males and 6 females) with a recent diagnosis of colorectal cancer and no signs of metastases and 12 healthy volunteers matched for age and gender were enrolled in the study. Colorectal cancer patients had histologically proven adenocarcinomas in biopsies taken during sigmo- or colonoscopy, and negative abdominal, chest and brain computed tomography scans for signs of metastases. They had not undergone any therapeutic intervention (surgery, chemotherapy or radiation therapy) before the time of blood sampling. Exclusion criteria were concomitant severe systemic diseases (diabetes mellitus, rheumatic diseases and cardiorespiratory insufficiency) or treatment during the last month with any medication known to affect oxidative stress parameters (vitamins C and E, allopurinol, N-acetyl-cysteine, corticosteroids, non-steroid anti-inflammatory drugs). Also patients and controls should have been non-smokers. During blood drawing no tourniquet constriction was used in order to minimize potentially enhanced oxidative stress induced by an ischaemia-reperfusion manoeuvre. All samples were taken using plastic syringes, and blood samples for oxidative stress measurements were placed in EDTA-containing tubes and immediately processed. The study was approved by the Ethics Committee of Patras University Hospital, Patras, Greece. Written informed consent was obtained from all subjects enrolled in the study.

### Blood treatment for oxidative stress measurements

Whole blood samples (0.2 ml) were brought to 300 µM Hydroethidine (HE) [O_2_^-^· specific trap] by the addition of 2 µl 30 mΜ ΗΕ stock [in 100% dimethyl sulfoxide (DMSO)], and incubated at 37°C for 30 min. The HE-incubated whole blood was then centrifuged at 3,000 *g* for 5 min in order to separate plasma from blood cells. Blood plasma was treated for O_2_^-^· and protein determination. In another experiment, plasma was firstly separated from whole blood, then incubated with HE at 37°C for 30 min and processed for O_2_^-^· determination. For TBARS measurement, 0.2 ml HE-untreated whole blood was used because HE interferes with the TBARS assay (9).

### O_2_^-^· assay

O_2_^-^· was assessed by application of a new ultrasensitive fluorescent assay, as previously described (7, 8, 10). O_2_^-^· concentration was expressed in pmole mg^-1^ protein (in 30 min).

### TBARS assay

Plasma samples (approx. 50 µl) were assayed by a modified TBA-based method as described previously (10).

### Protein concentration assay

Protein in approximately 400-fold diluted plasma sample was determined by a modification of a Coomassie Brilliant Blue-based method as described previously (10).

### Statistical analysis

Statistical analysis was performed using the SPSS statistical package (SPSS Inc, 2001, Release 11.0.0, USA). Results are expressed as mean ± SD and data were analyzed by non-parametric Mann-Whitney *U* test. A *P* value of less than 0.05 was considered significant.

## Results

**Figure 1:**
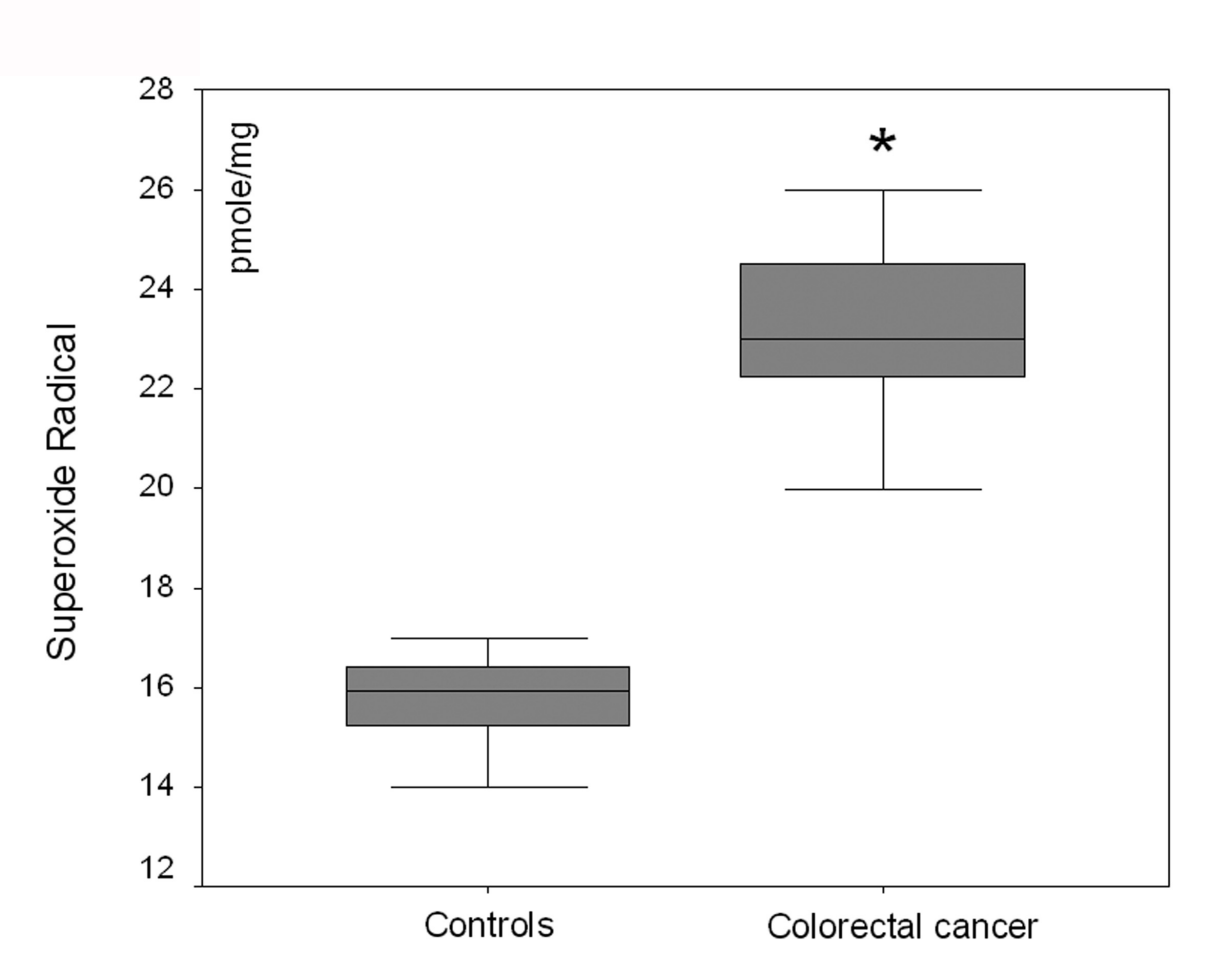
O_2_^-^· levels in the plasma fraction of whole blood in healthy volunteers and patients with non-metastatic colorectal cancer. *P<0.001 as compared to controls.

**Figure 2:**
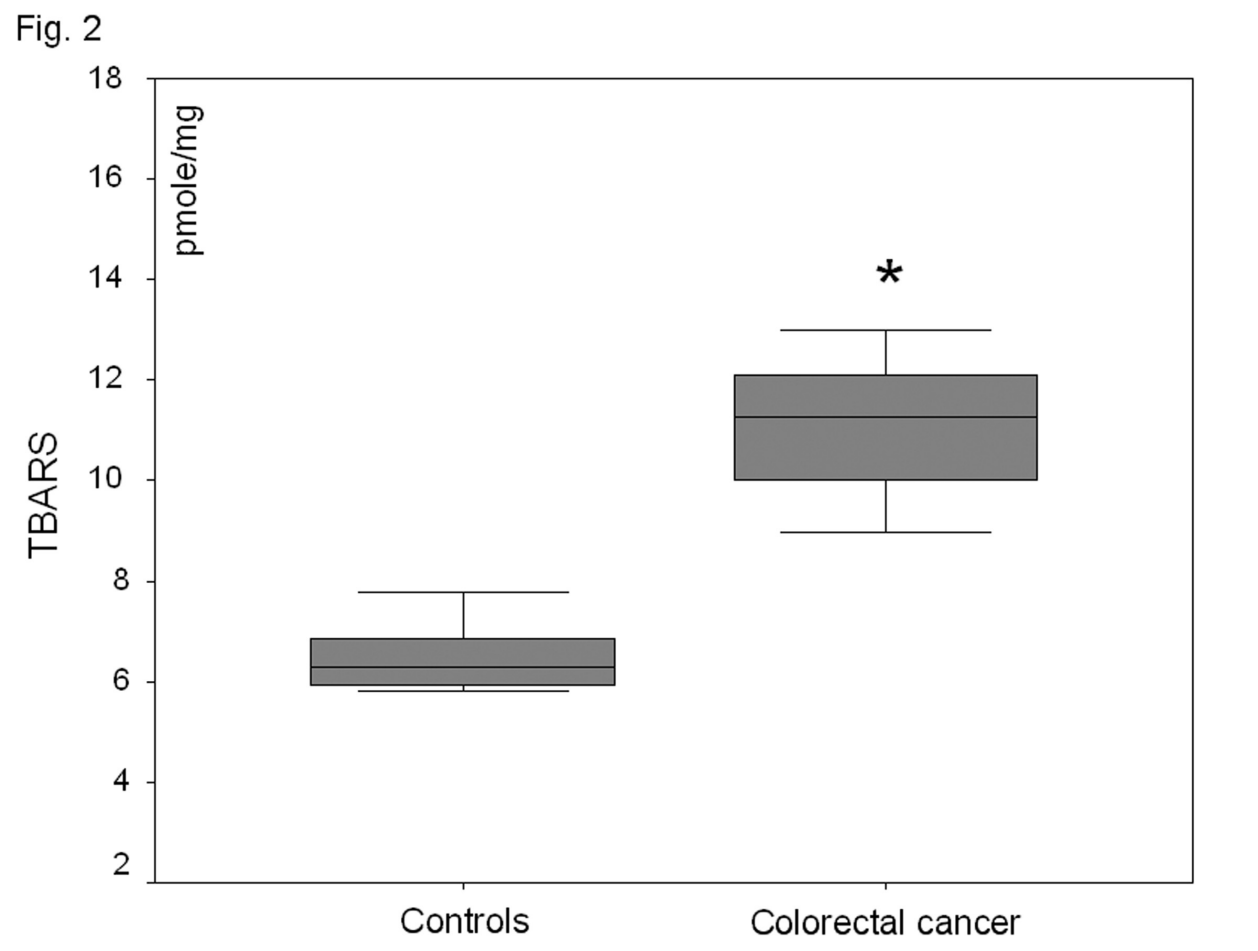
Plasma lipid peroxide (TBARS) levels in healthy-controls and patients with non-metastatic colorectal cancer. *P<0.001 as compared to controls.

In the plasma fraction of the HE-treated whole blood, there was a significant increase (47%) of O_2_^-^· levels in colorectal carcinoma patients as compared to healthy volunteers (P < 0.001) (Fig. 1). When plasma was firstly separated from whole blood and then treated with HE, no O_2_^-^· was detected in both groups. Plasma TBARS levels were increased by 81% in colorectal carcinoma patient as compared to controls (P<0.001) (Fig. 2).

## Discussion

Colorectal cancer is a major global health problem and the fourth most common cause of cancer death worldwide(11). Its aetiology is still under investigation, but a growing body of evidence has suggested that oxidative stress plays an important role in the molecular mechanism of colorectal cancer(4, 6, 12-15). Colorectal cancer is generally assumed to be initiated by environmental genotoxic agents causing cellular overproduction of reactive oxygen species (ROS). As a consequence, extensive oxyradical-mediated damage can cause genetic alterations required for neoplastic progression and lead to a cycle of cell death and regeneration (2, 15).

To the best of our knowledge, this is the first study directly assessing O_2_·^-^ in the blood of patients with colorectal cancer. Our results demonstrate a significant increase of O_2_·^-^ in the plasma of patients with non-metastatic colorectal cancer as compared to healthy controls. This is a direct proof that patients with colorectal cancer, even at an early stage, are exposed to systemic oxidative stress. Moreover, oxidative stress was also shown indirectly by the 81% increase of the lipid peroxidation marker TBARS. Those aldehydic decomposition end products of lipid peroxides found increased in the plasma of colorectal carcinoma patients might have been formed (a) by the direct oxidative attack of serum blood lipids by O_2_^-^· (and other ROS derived from O_2_^-^·, such as hydroxyl radical) (1), and (b) locally in the cancerous tissue by increased ROS formation and then entering the systemic circulation and transported through blood to remote sites (14, 16).

Regarding the potential sources of increased plasma O_2_^-^· detected *ex vivo* in patients with non-metastatic colorectal cancer, there are some important points we would like to highlight. Firstly, increased O_2_·^-^ measured in the plasma of our patients could not have been the result of its potential increased production locally at the site of tumour growth (14), given its very short half-life (1 µs) and its inability to cross cell membranes (1). Secondly, O_2_^-^· was detected *ex vivo* only in the plasma fraction of HE-treated whole blood and not in the initially separated and then HE-treated plasma. This finding suggests that increased plasma O_2_^-^· in patients with colorectal cancer is most likely generated by blood cells and not by a source in the plasma per se (e.g. an activated soluble enzyme present in the plasma of patients with cancer). Previous studies have shown that in cancer O_2_·^-^ may be overproduced by: (a) activated granulocytes (17), (b) macrophages and natural-killer cells activated to react against the tumour (18), or (c) by malignant cells (19). Given that in the present study our patients had a non-metastatic cancer, which means that malignant cells are not possibly present in the systemic circulation, we are tempting to speculate that increased plasma O_2_·^-^ in those patients is possibly the result of its overproduction by activated circulating immune cells.

In conclusion, the present study shows that colorectal cancer, even at early (non-metastatic) stages, induces systemic oxidative stress as evidenced by increased O_2_·^-^ levels measured in plasma. Given the important role of oxidative stress in carcinogenesis and the fact that O_2_·^-^ is considered its primary parameter, our findings if confirmed in larger studies might establish the potential validity of O_2_·^-^ as a new biomarker for colorectal cancer.
